# An approach to persons who are not willing to engage in behavioural change

**DOI:** 10.4102/safp.v66i1.5874

**Published:** 2024-08-29

**Authors:** Olufemi B. Omole, Deidré Pretorius, Klaus B. von Pressentin

**Affiliations:** 1 Division of Family Medicine, Department of Family Medicine and Primary Care, University of the Witwatersrand, Johannesburg, South Africa; 2 Department of Family Medicine and Primary Care, Faculty of Health Sciences, University of the Witwatersrand, Johannesburg, South Africa; 3 Department of Family, Community and Emergency Care, Faculty of Health Sciences, University of Cape Town, Cape Town, South Africa

**Keywords:** unwilling persons, precontemplation, lifestyle behaviours, counselling, behavioural change, 5Rs approach

## Abstract

With its unique position, primary health care (PHC) can provide health promotion and prevention services, including lifestyle behavioural counselling. Unhealthy lifestyle behaviours are very prevalent among patients attending PHC, with many patients unwilling to change or in the precontemplation stage. While patients in the contemplation stage are better managed using the 5As approach of motivational interviewing counselling, those unwilling or not ready for change necessitate a different approach, such as the 5Rs of motivational interviewing (MI) counselling. The 5Rs MI approach holds promise in motivating unwilling individuals to consider embarking on the journey of behavioural change. The 5Rs approach is not a stand-alone checklist of tasks implemented in isolation but is best integrated within a theoretical behavioural change framework. Of the four health-related behavioural change theoretical frameworks that are frequently used, the transtheoretical stages of the change model are the most used. This continued professional development article provides a summary review of the literature on behavioural change theories as they apply to lifestyle health behaviour change and presents the 5Rs approach as a feasible and practical approach to manage patients who are unwilling to change or in the precontemplation stage. This offers a beacon of hope for improved patient outcomes in a PHC system saddled with high prevalence of modifiable unhealthy lifestyle behaviours.

## Introduction

Unhealthy lifestyle behaviours abound among patients in South African primary health care (PHC).^1^ Evidence on the number of patients who are unwilling or not contemplating health-related behaviour change is scarce except for tobacco use, as most of them would not even present to healthcare services. For example, we know that one in seven adult smokers in the United States never plans to quit (based on an analysis of pooled data from the 2015 and 2016 nationally representative Tobacco Products and Risk Perceptions Surveys). Compared to others, these smokers are less likely to have tried any cessation intervention, suggesting that they may be unwilling to engage in any discussion on making a quit attempt.^2^ The proportions of overweight or obese and physically inactive persons who are unwilling to lose weight or engage in regular physical activities are unknown. Also, to motivate a patient to adopt a healthier diet and increase physical activities, the clinician must require an understanding of the unique perceptions, experiences and context of the patient, as well as assist the patient in achieving a balance between their feelings of shame, guilt and pride, before the ‘change talk’ can produce a favourable outcome.^3^ Even when patients have taken responsibility for their own behaviour, some time may lapse before help is sought, for example, it can take an average of 6 years (median of 3 years) before obese persons will approach a doctor for help.^4^ For many patients, the behaviour ‘change talk’ is never initiated, and when initiated, it is neither sustained nor actioned for the desired behaviour. Thirty-two per cent of patients in a Swedish survey of 52 595 patients, confirmed discussing lifestyle changes with their doctors and only 39.2% of them made behaviour change decisions.^5^ Similarly, in a review of 42 studies that randomised a total of 24 057 participants, doctors motivated change for decreased alcohol use in 55% and 39% of primary care and emergency rooms consultations, respectively.^6^ In a South African study in PHC, at most about 35% of tobacco users were offered brief advice at any visit in the last 12 months.^7^ Unfortunately, a large number of patients will not adhere to prescribed behaviour change strategies despite the lure of better health, and thus, it is important to appreciate that behaviour change is not a once-off magic bullet but an ongoing process that requires the PHC clinician to view every clinical encounter as an opportunity to initiate or reinforce motivation for behavioural change.^8^

Primary health care is well placed to offer health promotion and prevention services both opportunistically and to patients with identified health risks. Many of these services will involve counselling patients for a healthier lifestyle using behaviour change theories.^9^ The most used frameworks for behaviour change include the following:

- The transtheoretical stage of change model was proposed by Prochaska and DiClemente and is the most used of all the theories.^9,10^ Motivational interviewing (MI) enhances the patient–doctor relationship and a systematic review (72 studies) found that MI outperformed traditional advice in 80% of studies.^11^ The MI is a collaborative, person-centred form of guiding to elicit and strengthen motivation for change.^10,11,12^ It posits that people go through six stages of change from precontemplation through contemplation, preparation for change, change, maintenance and, if unsuccessful, a stage of relapse. The main task of the clinician using MI is to identify which stage the patient is in and motivate them to shift towards a desired change in behaviour. Fundamental principles include expressing empathy (seeing issues through the patient’s worldview), supporting patient self-efficacy (believing that the patient has an inherent capability to change successfully), rolling with resistance (when the patient experiences conflict between their views of the problem and solutions and those of the clinician) and creating discrepancy (so that the patient may perceive a mismatch between their current position and where they need to be, based on their values and self-identified goals).^12,13^ Limitations of this model include ignoring the social contexts in which change occurs, not having a time limit on how long a patient can be in a stage, and assumptions that once motivated, patients can always make informed decisions. The ‘spirit’ of MI involves collaboration (instead of confrontation), evocation (rather than imposing) and autonomy rather than authority.^12,13^
- Rosenstock developed the health belief model in 1966.^14,15^ This model posits that avoiding risky behaviour depends on the patient’s perceived susceptibility and severity, perceived benefits (of engaging in change) and perceived barriers (to change), cues to action and self-efficacy.
- The social cognitive theory was developed by Bandura in 1986.^16^ It posits that a behaviour change is a product of the interaction of personal factors, cognitive processes, and environmental or contextual influences. Primary constructs of this theory include observational learning, reinforcement, self-control and self-efficacy.
- The social ecological model was proposed in 2006 by Panter-Brick, and it posits that health behaviours are influenced by individual (knowledge, attitude, skills), interpersonal (social network), organisational (environmental, ethos) and community (culture, values and norms) level factors and public policy.^17^

While several behaviour theories and counselling frameworks facilitate and maintain behaviour change,^10^ doctors often struggle to initiate the discussion on behavioural change, especially when patients are not contemplating change and would instead engage in ‘sustain talk’.

This article briefly reviews relevant literature on lifestyle behaviour changes and common health-related behavioural change theories. It proposes the 5Rs as a viable and patient-centred approach to motivate patients who are not ready to engage in behaviour change.

## The 5Rs – Motivating patients not ready for lifestyle change

Doctors are familiar with the 5As approach (assess, advise, agree, assist and arrange)^13^ but may become challenged when they encounter patients unwilling to change or who have never considered a behaviour change. In the latter scenarios, the 5Rs approach (relevance, risks, rewards, roadblocks and repetition)^18,19^ offers hope as an evidence-based motivational method of exploring patients’ continuing engagement in unhealthy behaviour (sustain talk) and tilts their motivation to support decisions for change (change talk) (see Table 1). It is important to emphasise that the 5Rs approach is not implemented in isolation but within health-related behaviour change theories and frameworks such as the stages of change, social cognitive, social-ecological and health belief models. The 5Rs approach has been shown to be effective and encouraging ‘Learning Talk’ as a mediation pathway to improving smoking cessation outcomes in patients who is unwilling to participate in quit attempts.^20^

**TABLE 1:** Strategies for implementation of the 5Rs.

5Rs	Strategy	Example
Relevance	During history taking, the patient is already sensitised to the seriousness of the behaviour and condition when reflecting and answering the questions posed. After taking the history of the behaviour, the patient should be supported to think about change by reflecting on its meaning to the patient and what triggers or sustains it. The goal is to make the patient see the relevance of the behaviour to himself or herself and the need for change – how will change affect the patient, health outcomes, complication risks, family, or social context. The clinician can only do health education if the patient understands the disease and why the advice is relevant to the patient, and the change to desired health outcomes. In the same way, the doctor can only expect a patient to contemplate change if the patient is aware of the risks, hence the need to provide health education. If the patient thinks it is irrelevant, it is highly probable that the patient has not linked the risks correctly and, therefore, she or he struggles to see the relevance.	‘How do you think changing (avoid mentioning the specific habit. Keep it neutral first) will influence your health or illness?’ … ‘Will this also impact your family life or work?’ ‘Now that you know how your eating habits/exercise/smoking/alcohol consumption, etc influence your health and specifically the development of your disease, would you consider making a change?’ ‘I can see that you are struggling to see the relevance of this discussion. Will you want me to give you more information?’
Risks	Ask what the patient knows about the disease and its associated risks. The patient should reflect on risks that she or he is aware of before you start sharing information on other risks, including the consequences of continuing the risky behaviour – short and long term, for the patient, their significant others and their environment. Note: Do not withhold asking the above question because the patient has high educational attainment. Educational attainment is not equal to health literacy. Patients may have low health literacy and cannot link the consequences to the disease or pathology. Therefore, explain how behaviour leads to disease in lay terms.	‘What do you think about the harmful effects of your smoking/overeating/physical inactivity, etc, on your health?’ ‘Do you think it also affects your family/work negatively?’ ‘Do you need more information to understand the risks for your health and well-being?’
Rewards	Work with the available information from the patient and then ask questions about other aspects. Support the patient in exploring potential benefits of change including improved health status and quality of life, the prevention or delay of complications, etc. Focus on both short-term and long-term benefits.	‘Were you to make this change, tell me what benefits you may get?’ ‘If you permit, I can tell you of some benefits this change might bring to your family, work, finances, etc?’
Roadblocks	Explore barriers that the patient encountered in his or her previous quit attempts. Ask the patient to propose solutions that are feasible within their resources and skills. Later, ask if you could suggest other solutions. Affirm the patients’ suggestions are reasonable. Patients’ suggestions usually reflect their understanding of the problem, their perception of their own self-efficacy, and what is doable within their contexts and values.	‘Would you mind telling me some of the things that made your previous change attempt(s) difficult or impossible?’ ‘What do you suggest you could do differently?’ ‘Many patients who have succeeded received support from the multidisciplinary team. With your permission, can I refer you to a diabetes support group/Quitline in your area?’
Repetition	The healthcare professional needs to normalise relapse with the patient and motivate them to reengage the change attempt after a relapse. Usually, successful behavioural change occurs only after several change attempts.	‘It is a common experience that most smokers who quit do so after a few quit attempts. So, you should not feel abnormal about this attempt failing. What is more important is for you to see what went well and what did not and try again when you are better motivated and ready.’

*Source:* Kaner EFS, Beyer FR, Muirhead C, et al. Effectiveness of brief alcohol interventions in primary care populations. Cochrane Database Syst Rev. 2018;2:CD004148. https://doi.org/10.1002/14651858.CD004148.pub4; and, Prochaska JO, DiClemente CC. Toward a comprehensive model of change. In: Miller WR, Heather N, editors. Treating addictive behaviors: Processes of change. Plenum Press; p. 3–27. https://doi.org/10.1007/978-1-4613-2191-0_1

### Practical steps and key tasks of the 5Rs approach

It is vital to understand that the 5Rs are a conversation and not a fixed stepwise process, with the flow dictated by the doctor–patient interaction. Patient-centredness, good communication skills, active listening, empathy and a non-judgemental attitude will contribute to the success of this motivational intervention.^18,19^

- It is important to have an icebreaker to lighten the mood and create a welcoming environment. Ask for permission to explore the unhealthy behaviour together with the patient. Build rapport and trust with the patient as the counselling goes on by actively listening and employing a facilitatory and patient-centred approach, such as open-ended questions, affirming the patient, reflective listening, and summarising.^18,19^
- Encourage patient self-autonomy and let the patient know they are not pressured to change but encouraged to express their views without judgement. In addition, patient self-efficacy should be supported.^18,19^
- Agree on an agenda and the direction of engagement to objectify and guide the engagement. Identify the behaviour(s) that needs to be changed and focus on it or them.^18,19^

When the patient is comfortable and willing to focus, explore ‘sustain talk’, which refers to the patient’s views and reasons for continuing their unhealthy behaviours. The goal of exploring the ‘sustain talk’ is to identify how the patient expresses their precontemplation to help determine the best counselling approach. Patients unwilling to change may express their ‘sustain talk’ in several ways:

- Revelling: focusing only on their pleasant experiences of the unhealthy behaviour as they may not know yet what and how it is to experience adverse effects. Others would have experienced the negative effects but may normalise them, believing they deserve them. The clinician’s role is to provide non-judgemental feedback on health risks and the negative consequences to enable the patient to think and reflect on the reality of the consequences of the unhealthy behaviours.
- Reluctance: expressing a lack of knowledge about the problems that can arise from unhealthy behaviour and the impact of healthier behavioural change. Clinicians should provide non-judgemental reassurance that living without unhealthy behaviour and avoiding negative consequences is possible and beneficial.
- Rebellion: expressing fear of losing control of their lives, having invested heavily into the behaviour to be changed. The clinician should support the patient in making positive choices and taking responsibility rather than resisting because of perceived pressure from the clinician. One way to support is to ask questions to evoke the patient’s understanding and his or her internal locus of control, rather than making statements to pressure or scare the person to comply.
- Resignation: feeling powerless, hopeless and overwhelmed by the need to change or from past failures to change that may be haunting them. The clinician should support the patient in becoming optimistic about their self-efficacy to change, exploring barriers to change, acknowledging any past successes at change, and providing information on the usefulness of treatments and linkage to support groups, if available. The issue of resources needed for change should be explored, although unhealthy behaviours are also associated with ‘wasteful’ use of resources that could be re-channelled into facilitating desired change.
- Rationalisation: displaying an attitude of ‘I know it all’ and stating that unhealthy behaviour is other people’s problem, not theirs. Clinicians should use double reflection to acknowledge patients’ comments and highlight their concerns/reservations.^18,19^

## The key tasks of the 5Rs approach

**FIGURE 1:**
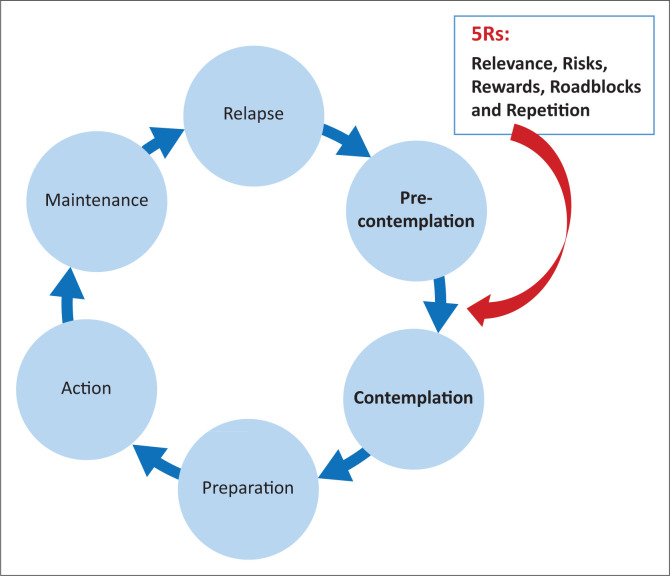
Stages of change model and the 5Rs.

According to Prochaska and DiClemente’s stages of change model, ‘… change is not a linear progression through the stages; rather, most clients move through the stages of change in a spiral pattern’.^9^ Figure 1 indicates where the 5Rs belong in the cycle of change.

Patients not contemplating behavioural change require a different approach than those contemplating. Clinicians often get frustrated with these patients because they need to prepare for change and sometimes perceive that they need to be more serious about their health. However, giving up a lifestyle behaviour can be emotionally challenging and may mimic the loss of a significant person.^21^ Also, pushing patients to commit to change before they are ready may exacerbate their defensive mode and make them less cooperative.

The 5Rs effectively increase a patient’s motivation and allow them to express their motivation for behaviour change in their own words.^6^ They also enable clinicians to tailor their responses to meet the patient’s needs (Table 1)^6,9^:

- Make the health risk relevant: To make the health risk relevant, the clinician should encourage the patient to express why the behaviour is personally relevant to the patient’s health, family or psychosocial context. For example, making a smoking quit attempt because there are children at home, for an asthmatic partner, or for a person with prediabetes losing weight to delay or avoid diabetes and its long-term complications.
- Explore the risk of continuing the behaviour: The clinicians should ask the patient to identify potential negative consequences of continuing the undesirable behaviour (e.g., smoking, physical inactivity, etc.) and affirm the patient by providing evidence in support of the patient’s correct views (e.g., that continuing cigarette smoking will increase the risk of stroke and heart attack) and address incorrect views (e.g., changing from regular cigarette to low tar cigarette). Both short- and long-term risks need to be explored.
- Explore the rewards of behaviour change: Ask the patient to identify potential benefits of the proposed change, for example, fewer asthma attacks and savings on cigarettes from stopping smoking. The aim is to support the patient in seeing the significant benefits and health impact of the behaviour change.
- Explore roadblocks or barriers: Most patients who engage in unhealthy behaviours would have made prior attempts at changing.^22^ Ask the patient to identify barriers to change in previous attempts, acknowledge them and attempt to provide problem-solving counselling to assist them in generating solutions. Where patients make reasonable suggestions, affirm and support them. Patients’ suggestions usually reflect their understanding of the problem, their perception of self-efficacy, and what is doable within their social context. Where the clinician is ill-equipped, referrals to members of the multidisciplinary team or specialised services should be made to provide feasible solutions to identified barriers, for example, the national smoking quit line or useful mobile apps.^18,23^
- Repetition: Most successful behaviour changes occur after several cycles of change. Clinicians need to assist patients in seeing that relapse is common. The clinician should motivate the patient to use renewed attempts to build on past successes and solve the existing or new roadblocks. Normalise the need to repeat the cycle of behaviour change and the motivational intervention at every clinical encounter by an unmotivated patient in the correct spirit. Make this discussion brief but repeat the agenda of the clinic visit if both parties agree.

## Challenges that may persist despite employing the 5Rs

Doctors must be aware that patients often make subjective change appraisals based on their social structures that often reward personal significance and the notion to favour momentary temptations and not valued goals for health.^24,25^ Unhealthy lifestyle habits develop over time, and long-term benefit of change does not give immediate gratification and is thus difficult to sustain.^21,25^ As behaviour change can be self-initiated (internal locus of control) or externally motivated (family, healthcare professional), impulse conflict, as well as social conflict can be triggered that can compromise the intention to change.^24^ Furthermore, stress and cravings can be a barrier to change despite the best intentions.^7,25^

## Summary

- While most patients needing lifestyle behaviour change in primary care will be contemplating, many will be unwilling to engage in the change talk for various reasons.
- The 5As approach can support patients willing to change. Still, those who are unwilling or not contemplating can benefit from the 5Rs approach – exploring the relevance of the risks to place the patient on the risk spectrum, the negative consequences of sustaining the risk, the rewards of change, the roadblocks or barriers, and repeating in case of relapse.
- There are many health-related behavioural theories, but the transtheoretical stages of change model utilising MI remain the most effective. Skills in the principles and spirit of MI allow for the effective use of this approach. Whichever framework or model is used should not be an imposition of change. Instead, the aim should be collaboration that empowers the patient’s autonomy to decide to change.

In conclusion, behavioural change is not an easy endeavour, and not all patients are willing to embark on the journey for many reasons. The clinician must be capacitated to know what to do for the latter category of patients. The 5Rs approach is an evidence-based approach to supporting and motivating unwilling patients to embark on the journey of behavioural change.
